# Supplementary material for: Assessing Trade-Offs and Optimal Ranges of Density for Life Expectancy and 12 Causes of Mortality in Metro Vancouver, Canada, 1990–2016
Source: Int J Environ Res Public Health. 2022 Mar 2;19(5):2900. doi: 10.3390/ijerph19052900 (PMC8910136; doi:10.3390/ijerph19052900)
Supplement: Supplementary file 1 [file ijerph-19-02900-s001.zip › ijerph-1573183-SI.pdf]

Supplementary materials

Figure S1. Trend Analysis in Metro Vancouver (all CTs).

Life expectancy:

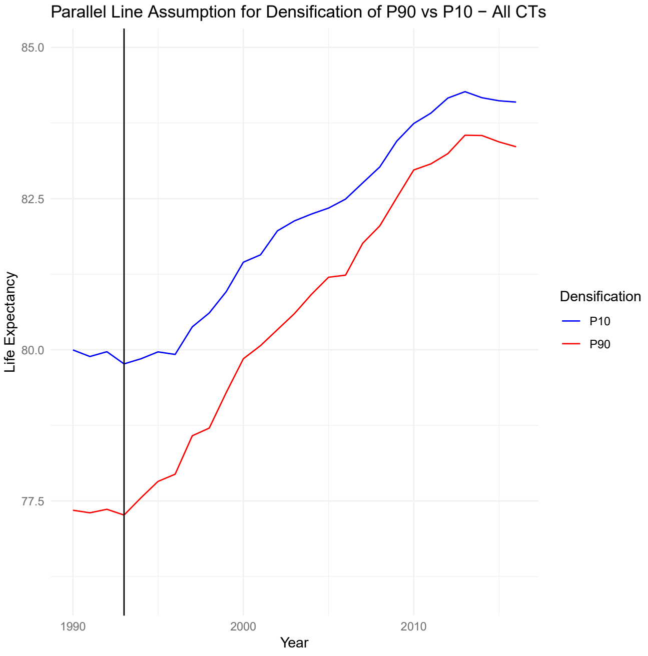

Cause-specific mortality:

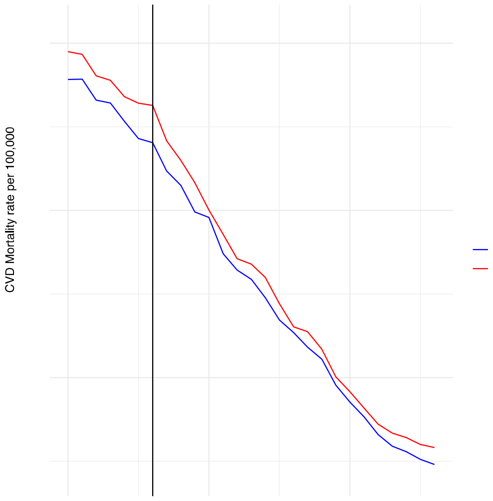

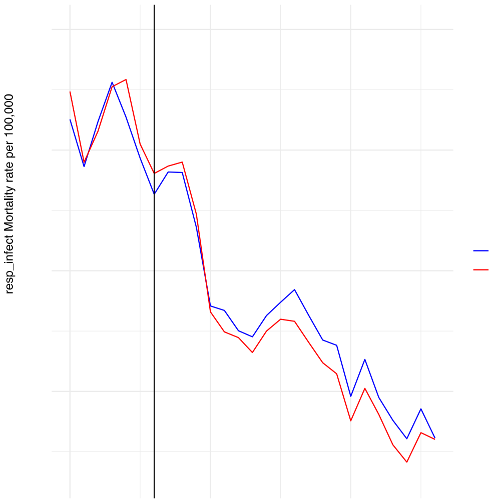

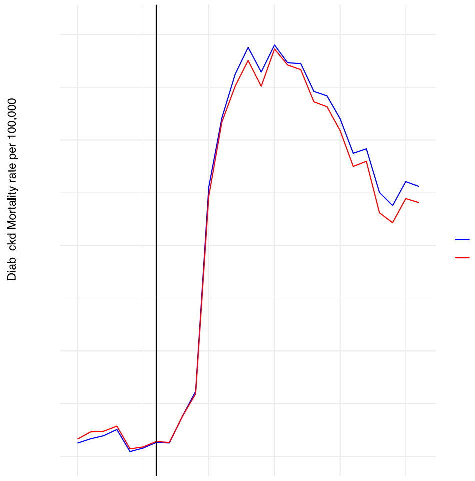
Diab_ckd Mortality rate per 100,000

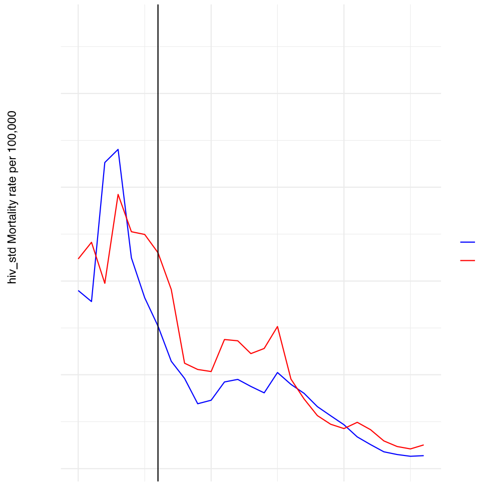
hiv_std Mortality rate per 100,000

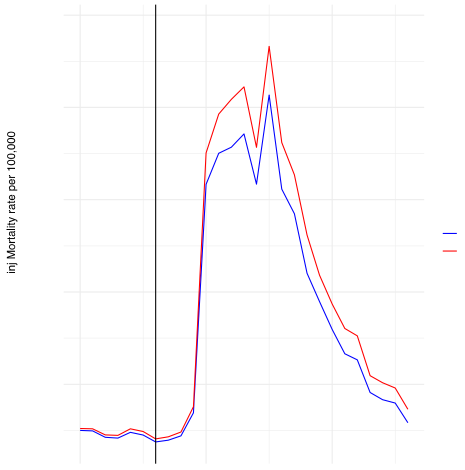
inj Mortality rate per 100,000

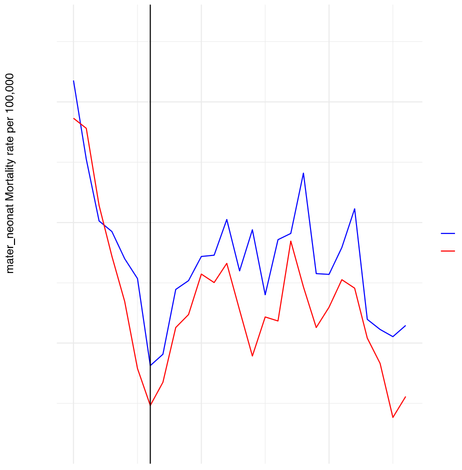
mater_neonat Mortality rate per 100,000

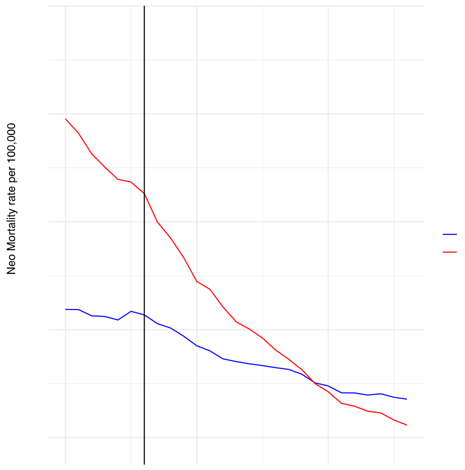
Neo Mortality rate per 100,000

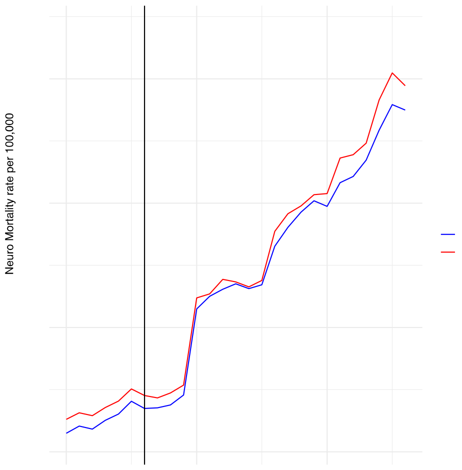
Neuro Mortality rate per 100,000

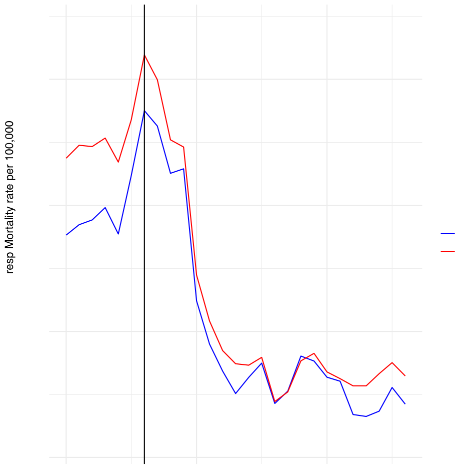
resp Mortality rate per 100,000

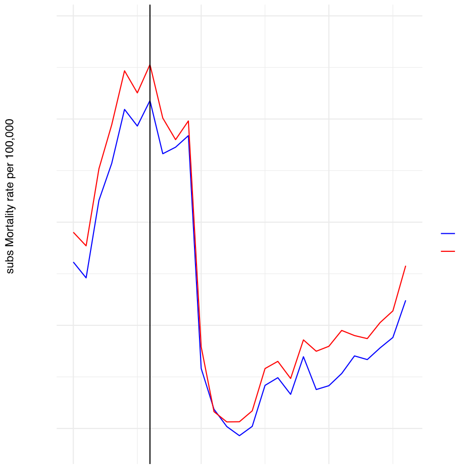
subs Mortality rate per 100,000

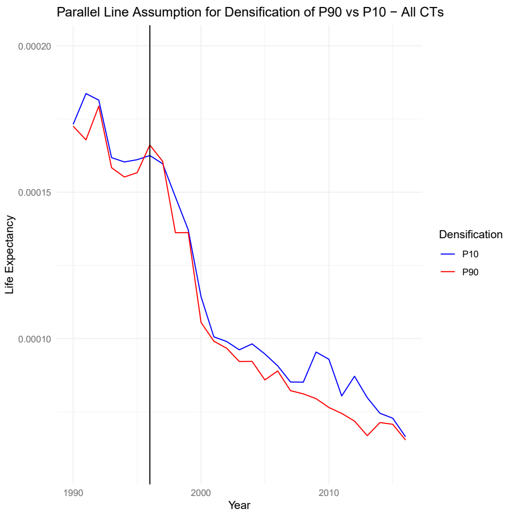
Parallel Line Assumption for Densification of P90 vs P10 − All CTs
0.00020
0.00015
0.00010
Life Expectancy
1990
2000
2010
Year
Densification
P10
P90

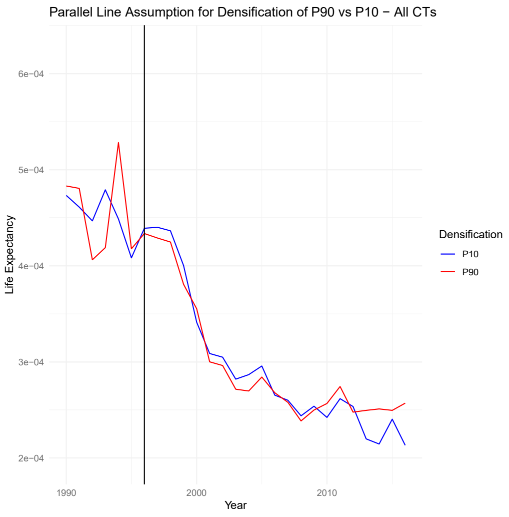
Parallel Line Assumption for Densification of P90 vs P10 − All CTs
6e−04
5e−04
4e−04
3e−04
2e−04
Life Expectancy
1990
2000
2010
Year
Densification
P10
P90

Figure S2. Treatment and control groups in the difference-in-differences analysis in model 1 (all CTs), 1990-2016, Metro Vancouver (T: treatment; C: control).

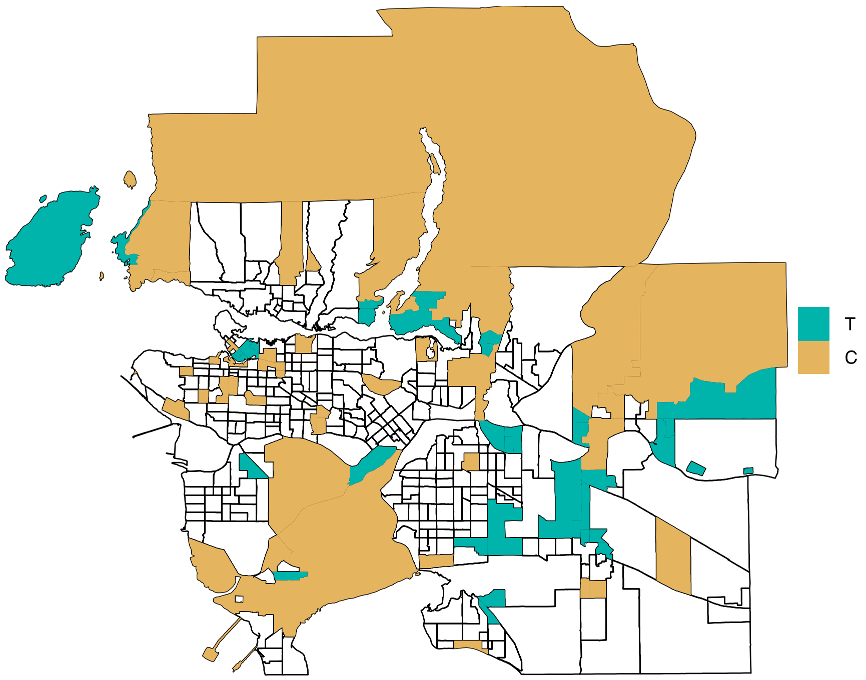

Figure S3. Optimal ranges of density for cause-specific mortality rates.

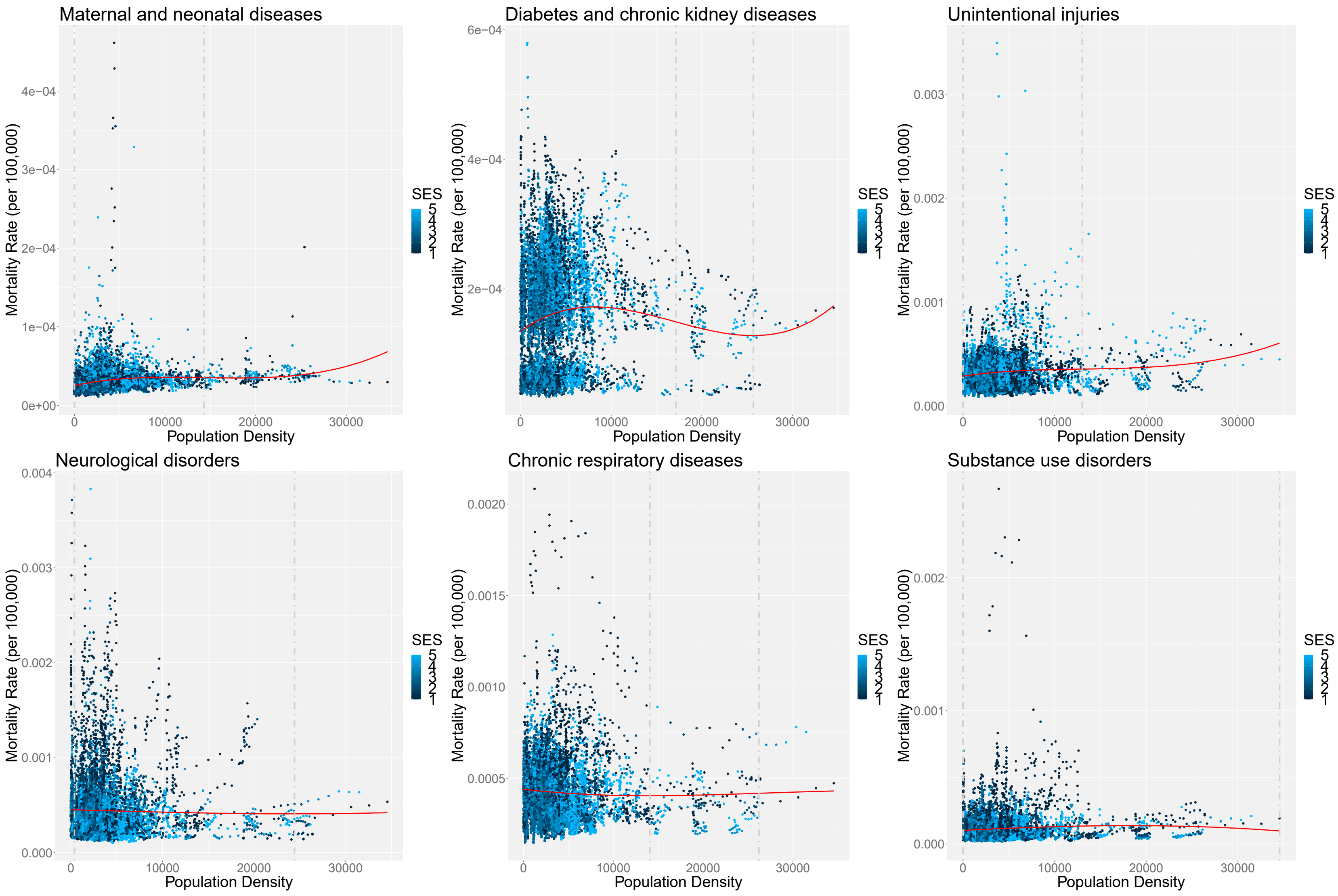

Figure S4. Optimal ranges of density LE and cause-specific mortalities for 2016 only.

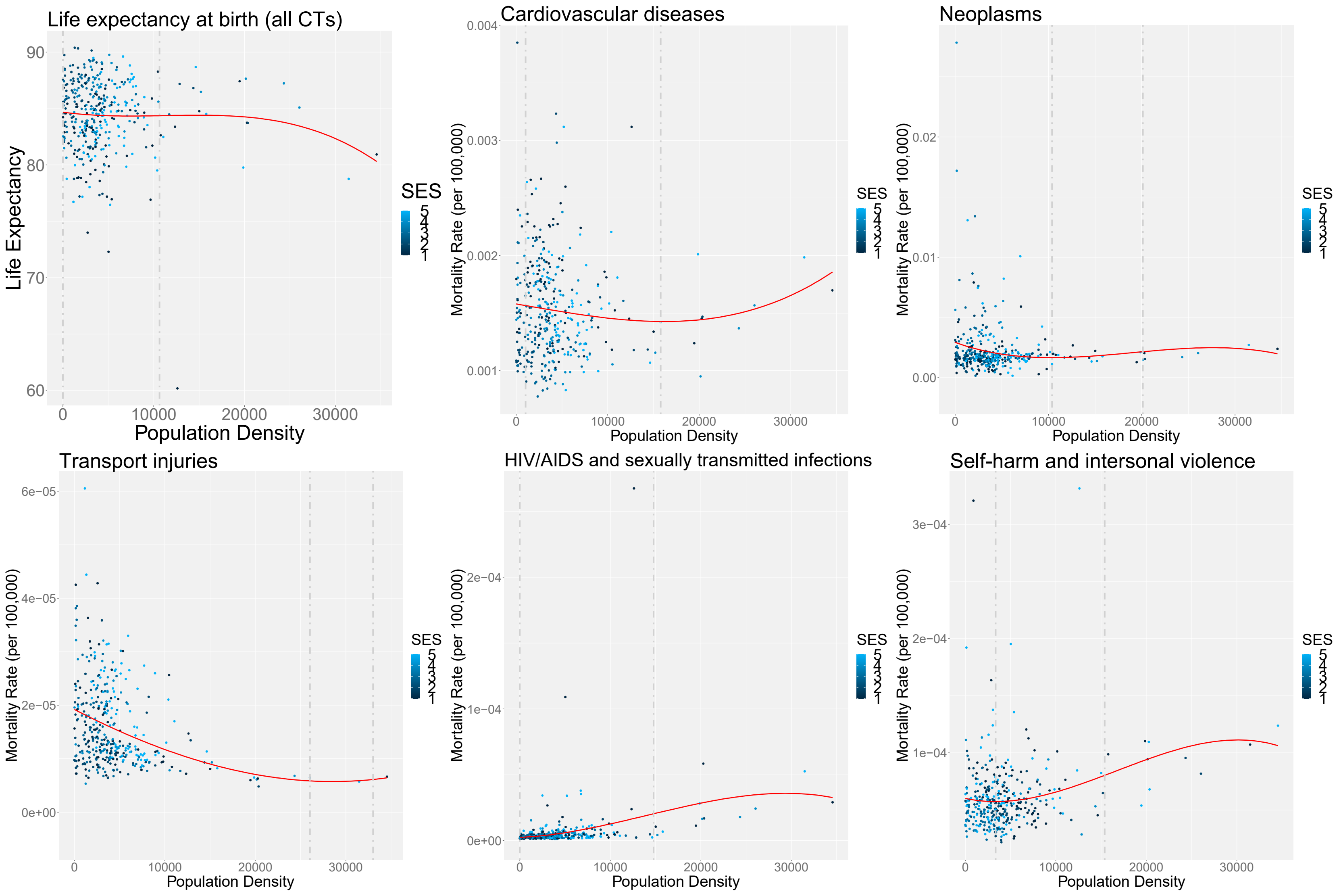

Figure S5. First order derivative of mortality functions (dY: difference of Y, dX: difference of X).

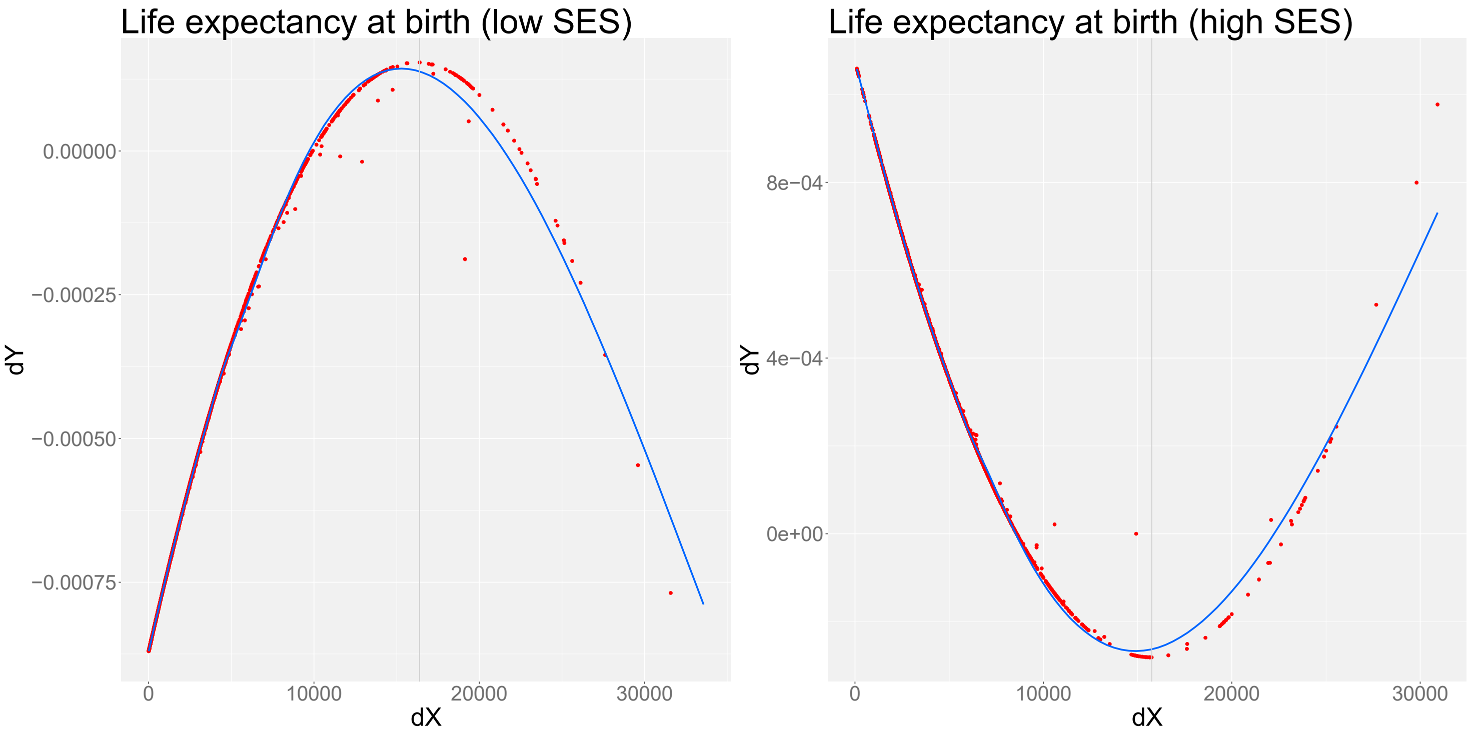

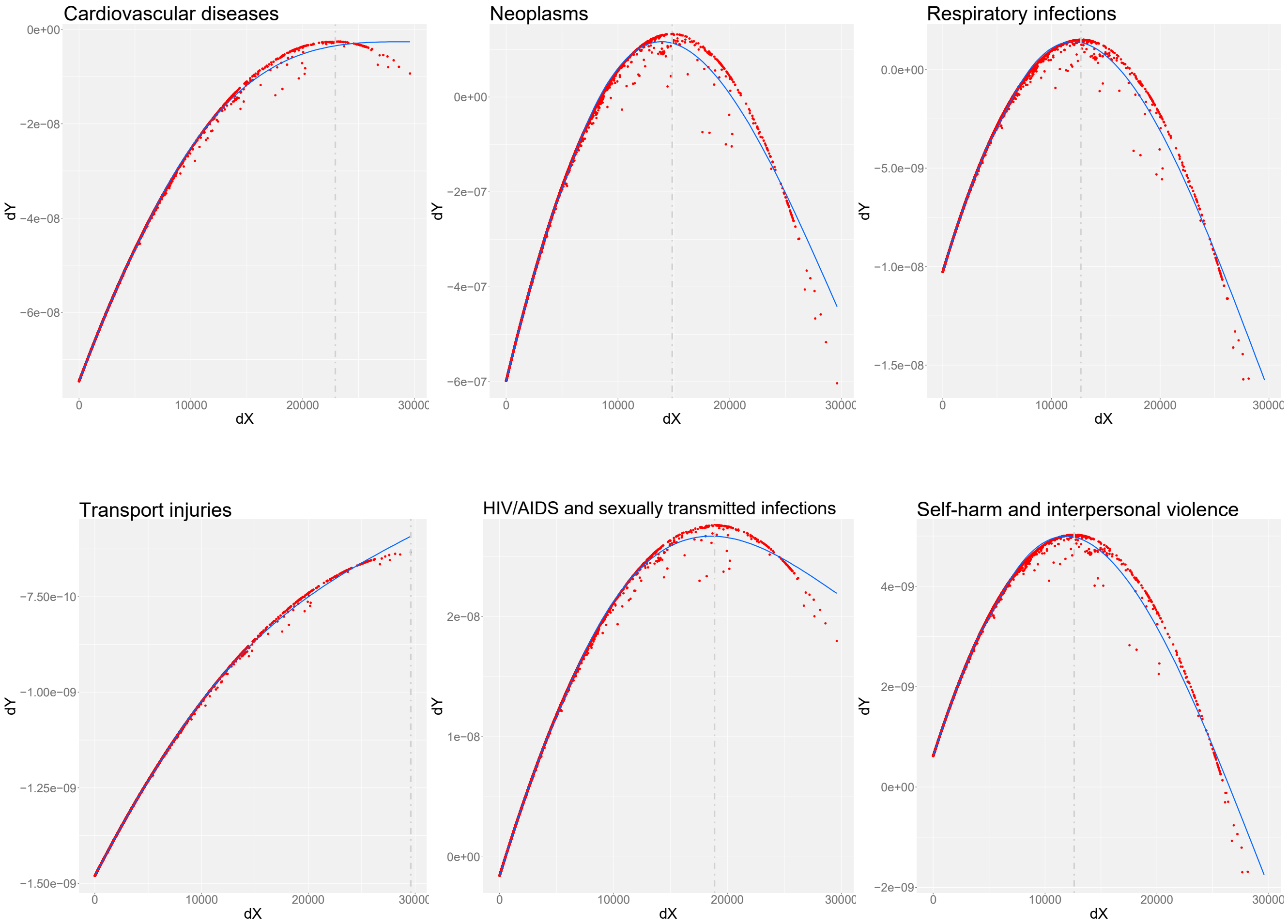
Cardiovascular diseases
0e+00
−2e−08
−4e−08
−6e−08
dY
0
10000
20000
30000
dX
Neoplasms
0e+00
−2e−07
−4e−07
−6e−07
dY
0
10000
20000
30000
dX
Respiratory infections
0.0e+00
−5.0e−09
−1.0e−08
−1.5e−08
dY
0
10000
20000
30000
dX
Transport injuries
−7.50e−10
−1.00e−09
−1.25e−09
−1.50e−09
dY
0
10000
20000
30000
dX
HIV/AIDS and sexually transmitted infections
2e−08
1e−08
0e+00
dY
0
10000
20000
30000
dX
Self-harm and interpersonal violence
4e−09
2e−09
0e+00
−2e−09
dY
0
10000
20000
30000
dX

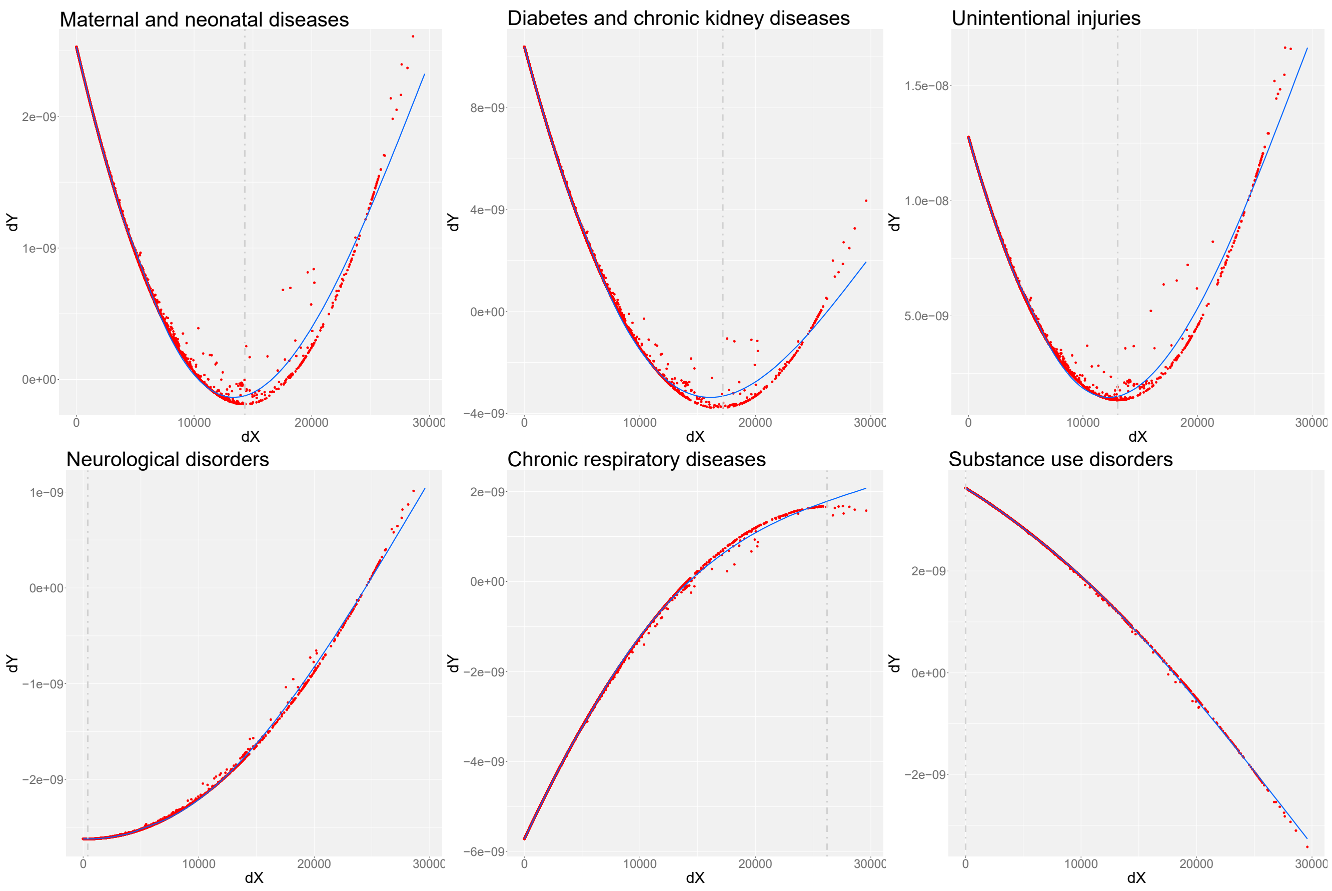

Maternal and neonatal diseases
Diabetes and chronic kidney diseases
Unintentional injuries
Neurological disorders
Chronic respiratory diseases
Substance use disorders
dY
dX
0
10000
20000
30000
2e−09
1e−09
0e+00
8e−09
4e−09
−4e−09
1.5e−08
1.0e−08
5.0e−09
−1e−09
−2e−09
−4e−09
−6e−09

Table S1. Difference-in-Differences Estimates of the Impact of Densification levels on Mortality Outcomes, Metro Vancouver, British Columbia, 1990-2016 – All CTs.

	All CTs				
**Outcome**	**β**	**95% LB**	**95% UB**	**SE**	R^2
Life expectancy[a]	-8.66e-06	-2.02e-04	1.94e4	9.84e-05	0.01
Cardiovascular diseases[b]	-1.26e-05	-2.85e-04	3.34e-05	7.8e-05	0.39
Diabetes mellitus and chronic kidney diseases	-8.60e-06	-2.21e-05	4.91e-06	6.88e-06	0.43
Neoplasms[b]	**-1.2e-03****	-1.67e-03	-7.30e-04	2.4e-04	0.06
HIV and sexually transmitted infections[b]	-5.26e-05	-1.13e-04	7.94e-06	3.09e-05	0.05
Maternal and neonatal diseases[b]	3.64e-07	-1.74e-06	2.47e-06	1.07e-07	0.02
Self-harm and interpersonal violence	-2.42e-06	-1.64e-05	1.15e-05	7.109e-06	0.18
Transport injuries[b]	**6.72e-06***	6.45e-08	1.34e-05	3.39e-06	0.24
Substance use disorders[b]	**-3.69e-05***	-6.95e-05	-4.31e-06	1.66e-05	0.19
Neurological diseases[b]	-1.39e-06	-6.00e-05	5.72e-05	2.99e-05	0.15
Chronic respiratory diseases	**-4.82e-05****	-8.45e-05	-1.17e-05	1.86e-05	0.12
Respiratory infections	-1.91e-05	-5.47e-05	1.66e-05	1.82e-05	0.15
Unintentional injuries	-6.29e-07	-4.53e-05	4.40e-05	2.28e-05	0.09

Abbreviation: SE, standard error; LB, lower boundary; UB, upper boundary
*P <0.05; **P <0.01
[a]Life expectancy at birth
[b]Mortality rate per 100,000

**Table S2. Difference-in-Differences Estimates of the Impact of Densification levels on Mortality Outcomes, Metro Vancouver, British Columbia, 1990-2016 – Low SES CTs.**					
			**Low SES CTs**		
**Outcome**	**β**	**95% LB**	**95% UB**	**SE**	R^2
Life expectancy[a]	**3.34****	2.17	4.51	0.60	0.48
Cardiovascular diseases[b]	**3.67e-04***	4.13e-05	6.94e-04	1.67e-04	0.45
Diabetes mellitus and chronic kidney diseases	**-5.12e-06****	-8.14e-05	-2.10e-05	1.54e-05	0.57
Neoplasms[b]	**-1.51e-04***	-2.73e-03	-2.83e-04	6.22e-04	0.06
HIV and sexually transmitted infections[b]	-4.77e-05	6.84e-05	2.18e-04	1.26e-04	0.14
Maternal and neonatal diseases[b]	**6.78e-06***	8.48e-07	1.27e-05	3.02e-06	0.04
Self-harm and interpersonal violence	**4.14e-05****	1.57e-05	6.72e-05	1.31e-05	0.54
Transport injuries[b]	3.71e-06	-9.23e-06	1.66e-05	6.58e-06	0.23
Substance use disorders[b]	-6.63e-06	-5.61e-05	4.28e-05	2.52e-05	0.51
Neurological diseases[b]	-3.21e-08	-1.84e-04	1.84e-04	9.36e-05	0.17
Chronic respiratory diseases	-1.40e-05	-8.45e-05	5.65e-05	3.59e-05	0.19
Respiratory infections	-2.01e-05	-9.73e-05	5.71e-05	3.92e-05	0.56
Unintentional injuries	**1.20e-04****	4.97e-05	1.90e-04	3.56e-05	0.47

Abbreviation: SE, standard error; LB, lower boundary; UB, upper boundary
*P <0.05; **P <0.01
[a]Life expectancy at birth
[b]Mortality rate per 100,000

Table S3. Difference-in-Differences Estimates of the Impact of Densification levels on Mortality Outcomes, Metro Vancouver, British Columbia, 1990-2016 – High SES CTs.

	High SES CTs				
**Outcome**	**β**	**95% LB**	**95% UB**	**SE**	R^2
Life expectancy[a]	-0.85	-2.6	0.89	0.88	0.19
Cardiovascular diseases[b]	**-9.93e-04****	-1.27e-03	-7.21e-05	1.39e-04	0.47
Diabetes mellitus and chronic kidney diseases	-8.03e-06	-3.26e-05	1.66e-05	1.25e-05	0.48
Neoplasms[b]	-1.57e-03	-3.33e-03	1.19e-04	8.61e-04	3.71 e-03
HIV and sexually transmitted infections[b]	-3.60e-06	-3.00e-05	2.28e-05	1.34e-05	0.18
Maternal and neonatal diseases[b]	1.22e-06	-4.75e-06	7.20e-06	3.05e-06	0.02
Self-harm and interpersonal violence	**-3.35e-05****	-5.83e-05	-8.72e-06	1.27e-05	0.28
Transport injuries[b]	1.05e-05	-2.07e-06	2.31e-05	6.42e-06	0.25
Substance use disorders[b]	**-7.16e-05****	-9.09e-05	-5.22e-05	9.87e-06**	0.39
Neurological diseases[b]	**-1.01e-04****	-1.75e-04	-2.84e-05	3.75e-05**	0.15
Chronic respiratory diseases	**-6.90e-05****	-1.19e-04	-1.85e-05	2.57e-05**	0.21
Respiratory infections	**-1.84e-04****	-2.26e-04	-1.42e-04	2.13e-05**	0.11
Unintentional injuries	**-8.70e-05***	-1.72e-04	-1.82e-06	4.34e-05	0.14

Abbreviation: SE, standard error; LB, lower boundary; UB, upper boundary
*P <0.05; **P <0.01
[a]Life expectancy at birth
[b]Mortality rate per 100,000

Table S4. Density thresholds at the lowest mortality rate, inflection point, and range of optimal density for varying health outcomes (population per km2).

Outcome	Lowest mortality rate	Inflection point	Optimal Range
Life expectancy at birth	4.2	9368	4.2-9368
Cardiovascular diseases	34550	20955	20955-34550
Chronic respiratory diseases	16785	4.5	4.5-16785
Diabetes mellitus and kidney diseases	25870	17624	17624-25870
HIV and sexually transmitted infections	412	13801	412-13801
Maternal and neonatal disorders	4.3	14852	4.3-14852
Neoplasms	34550	14954	14954-34550
Neurological disorders	18878	9056	9056-18878
Respiratory infections	34550	14312	14312-34550
Self-harm and interpersonal violence	4.2	12606	4.2-12606
Substance use disorders	4.2	34550	4.2-34550
Transport injuries	34550	18625	18625-34550
Unintentional injuries	4.2	13024	4.2-13024
